# Outbreak of Beriberi among African Union Troops in Mogadishu, Somalia

**DOI:** 10.1371/journal.pone.0028345

**Published:** 2011-12-21

**Authors:** John T. Watson, Hassan El Bushra, Emmaculate J. Lebo, Godfrey Bwire, James Kiyengo, Gideon Emukule, Victor Omballa, John Tole, Muvunyi Zuberi, Robert F. Breiman, Mark A. Katz

**Affiliations:** 1 World Health Organization, Geneva, Switzerland; 2 World Health Organization, Regional Office for the Eastern Mediterranean, Cairo, Egypt; 3 Centers for Disease Control and Prevention, Nairobi, Kenya; 4 Uganda Defense Force (UDPF), Entebbe, Uganda; 5 Kenya Medical Research Institute, Nairobi, Kenya; 6 Aga Khan University Hospital, Nairobi, Kenya; 7 African Union Headquarters, Addis Ababa, Ethiopia; University of Maryland, United States of America

## Abstract

**Context and Objectives:**

In July 2009, WHO and partners were notified of a large outbreak of unknown illness, including deaths, among African Union (AU) soldiers in Mogadishu. Illnesses were characterized by peripheral edema, dyspnea, palpitations, and fever. Our objectives were to determine the cause of the outbreak, and to design and recommend control strategies.

**Design, Setting, and Participants:**

The illness was defined as acute onset of lower limb edema, with dyspnea, chest pain, palpitations, nausea, vomiting, abdominal pain, or headache. Investigations in Nairobi and Mogadishu included clinical, epidemiologic, environmental, and laboratory studies. A case-control study was performed to identify risk factors for illness.

**Results:**

From April 26, 2009 to May 1, 2010, 241 AU soldiers had lower limb edema and at least one additional symptom; four patients died. At least 52 soldiers were airlifted to hospitals in Kenya and Uganda. Four of 31 hospitalized patients in Kenya had right-sided heart failure with pulmonary hypertension. Initial laboratory investigations did not reveal hematologic, metabolic, infectious or toxicological abnormalities. Illness was associated with exclusive consumption of food provided to troops (not eating locally acquired foods) and a high level of insecurity (e.g., being exposed to enemy fire on a daily basis). Because the syndrome was clinically compatible with wet beriberi, thiamine was administered to ill soldiers, resulting in rapid and dramatic resolution. Blood samples taken from 16 cases prior to treatment showed increased levels of erythrocyte transketolase activation coefficient, consistent with thiamine deficiency. With mass thiamine supplementation for healthy troops, the number of subsequent beriberi cases decreased with no further deaths reported.

**Conclusions:**

An outbreak of wet beriberi caused by thiamine deficiency due to restricted diet occurred among soldiers in a modern, well-equipped army. Vigilance to ensure adequate micronutrient intake must be a priority in populations completely dependent upon nutritional support from external sources.

## Introduction

Since 1991, Somalia has lacked a functioning central government, resulting in extreme, longstanding insecurity. The continuing humanitarian crisis in Somalia has been called the world's worst by the UN [1], and is characterized by massive population displacement, frequent drought, widespread malnutrition, and fierce fighting among armed groups. In February 2007, the UN Security Council approved a peace support mandate (Resolution 1863) for the African Union Mission in Somalia (AMISOM). AMISOM soldiers are headquartered at the Base Camp adjacent to the Mogadishu airport. The force is well-equipped and consists of Burundian (N = 2100) and Ugandan (N = 3650) contingents stationed primarily at nine camps around Mogadishu.

The activity of AMISOM soldiers in Mogadishu is limited; repeated violent attacks, including mortar fire, gunfire, and roadside and suicide bomb attacks have made movement around the city difficult and dangerous [2]. Security constraints in Mogadishu severely limit the local procurement of fresh foods for the 5750 soldiers, limiting fruit, vegetable and meat consumption. On February 22, 2009, an attack in Mogadishu (at the time the worst attack to date) killed 11 AMISOM soldiers and injured 15, and resulted in a further tightening of security measures [3]. Interaction with the surrounding community, including formal and informal procurement of fresh food, declined even further.

### The Outbreak

On July 28, 2009, the World Health Organization (WHO) was notified by the African Union (AU) that 21 AMISOM soldiers in Mogadishu had become sick, and three had died, with an illness characterized by acute onset of peripheral edema, difficulty breathing, palpitations, and fever. WHO, together with the US Centers for Disease Control and Prevention, AU, AMISOM, and the Aga Khan University Hospital (AKUH-N), a private hospital in Nairobi, Kenya, initiated an investigation to determine the cause of the outbreak and to prevent further cases and deaths. This investigation was considered by all partners to constitute urgent disease control activity, and was viewed as public health practice and not research. A unanimous decision was made to proceed without delay to identify and control health risks. Ethical review was not deemed necessary for the investigation and control activities documented in this report. This study was exempt from Institutional Review Board approval as it is a description of an outbreak and response that had already occurred.

According to AMISOM doctors on the base in Mogadishu, the initial patients had been treated in Mogadishu for malaria and for leptospirosis without resolution of symptoms. Severe, unstable patients had been evacuated to AKUH-N for further care. No cases among the local population had been identified.

## Methods

AMISOM operates a level II hospital at Base Camp in Mogadishu, a facility which serves as a referral center for ill soldiers. Symptomatic soldiers were seen at the Base Camp facility, and the severely ill soldiers were airlifted to AKUH-N. For this investigation, a case was defined as any AU soldier with acute onset of lower limb edema, and at least one of the following additional signs or symptoms: dyspnea, chest pain, palpitations, nausea, vomiting, abdominal pain, or headache.

### Case-control study

We performed a case-control study among AMISOM troops in Mogadishu to identify specific risk factors for illness. We selected controls based on availability of asymptomatic soldiers for interview, and aimed to interview as many controls as possible given the short time available in the field. For every case and control, we administered a questionnaire collecting demographic information, rank and unit of military service, nationality, symptoms, information about type and location of foods consumed, sources of water, previous medical treatments, use of mosquito nets, use of insect repellants, self-reported HIV status, vaccination history, duration of stay in Mogadishu, exposure to gun and bomb fumes, job-related activities, and perceived stress.

Questionnaires were administered on the AU bases from August 19–21, 2009. Data were entered into a MS Access database. Bi-variate logistic regression analysis was performed. Odds ratios and 95% confidence intervals were calculated. Statistical analyses were performed with the use of SAS (version 9.1). P values of <0.05 were considered to be statistically significant.

### Clinical Investigation

We interviewed hospitalized soldiers who met the case definition and who had been transferred to AKUH-N from July 22–Aug 6, 2009, initially through unstructured interviews and later using a standardized questionnaire. For the patients admitted to intensive care who could not be interviewed, we collected information about clinical history from the attending physicians. We also reviewed medical charts for all patients and collected information about laboratory test results, other diagnostic testing, and clinical course using a standardized abstraction form.

### Laboratory Investigation

For soldiers who presented to AKUH-N we collected whole blood, serum, stool, nasopharyngeal (NP) and oropharyngeal (OP) swabs, and urine for testing at non-AKUH-N laboratories to investigate the presence of infectious diseases, toxic substances and nutritional deficiencies. Specific tests that were performed are described in Table 1. Samples were tested in laboratories at the CDC-Atlanta, CDC-Nairobi, Walter Reed Army Medical Center in Washington DC, the National Institute for Environmental Health Sciences in the US, and the National Influenza Center/Kenya Medical Research Institute-Nairobi for a variety of pathogens.

**Table 1:** Available laboratory results for cases referred for admission to AKUH-N (n = 33).

*Hematology Findings* 1	High	Low	Normal	Total
WBC Total	0	3	22	25
Red Cell Ct	0	3	22	25
Hemoglobin	0	5	27	32
Hematocrit	0	4	29	33
Platelet Ct	1	3	29	33
***Biochemisty*** 1				
Albumin	0	3	21	24
Bilirubin Serum	11	0	19	30
Bilirubin Direct	21	0	9	30
ALP	0	0	29	29
SGOT	5	0	27	32
SGPT	6	0	26	32
Gamma GT	6	0	24	30
Serum Potassium	2	5	26	33
Creatinine	13	0	20	33

*Pathogen Screening*	*Positive*	*Negative*	*Total*
Respiratory viral pathogens (PCR)^2^			
Influenza A and B	0	18	18
Respiratory Syncintial Virus	0	18	18
Human Metapneumovirus	0	18	18
Parainfluenza Virus	0	18	18
Brucella-PCR^3^	0	18	18
CCHF-PCR^2^	0	18	18
Chikungunya-IgM^2^	0	18	18
Dengue IgM^2^	11	7	18
Ebola(Zaire,Sudan,Bundibugyo)-PCR^2^	0	18	18
Ebola-Elisa Ag, IgM^2^	0	18	18
Hepatitis E- IgM/PCR^2^	7	11	18
LASSA^2^	0	18	18
Leptospira- IgM^3^	0	18	18
Leptospira- PCR^3^	0	18	18
Marburg- Elisa Ag,IgM^2^	0	18	18
Panentroviruses^4^	0	18	18
Ricketssia-PCR^3^	0	18	18
RVF IgM^2^	0	18	18
RVF PCR^2^	0	18	18
West Nile-IgM^5^	0	18	18
West Nile-PCR^5^	0	18	18
Yellow Fever –IgM^2^	0	18	18

1 Aga Khan Laboratories- Nairobi, Kenya.

2 Center for Disease Control and Prevention Laboratories-Nairobi, Kenya.

3 Kenya Medical Research Institute-Kisumu, Kenya.

4 Walter Reed Army Medical Laboratories-Nairobi, Kenya.

5 National Influenza Center, Nairobi, Kenya.

Blood specimens were tested for Vitamin B1 levels at the Medical Research Council Human Nutrition Laboratory in Cambridge, UK. Thiamine levels were determined by cobas and microplate assay of ETKAC (erythrocyte transketolase activation coefficient) performed in lysates [4].

### Environmental Investigation

At the AMISOM base in Mogadishu, we inspected the food preparation and cooking facilities and observed the process of serving and preparation of food for the troops. In addition, we collected samples of cooking oil for laboratory testing. Cooking oil and cooked food was tested for argemone alkaloids, due to the past association of this substance with outbreaks of pedal edema and right-sided heart failure among those exposed to contaminated cooking oil [5]. The testing was conducted at three laboratories: the Geneva Food Authority Control (Service de la Consommation et des Affaires Vétérinaires), Geneva Switzerland; the Department of Chemical Pathology of the National Health Laboratory Service, Johannesburg, South Africa; and at the Battelle Memorial Institute laboratory in Columbus, Ohio, USA, which is part of the National Toxicology Program of the US National Institute for Environmental Health Sciences.

## Results

**Figure 1:**
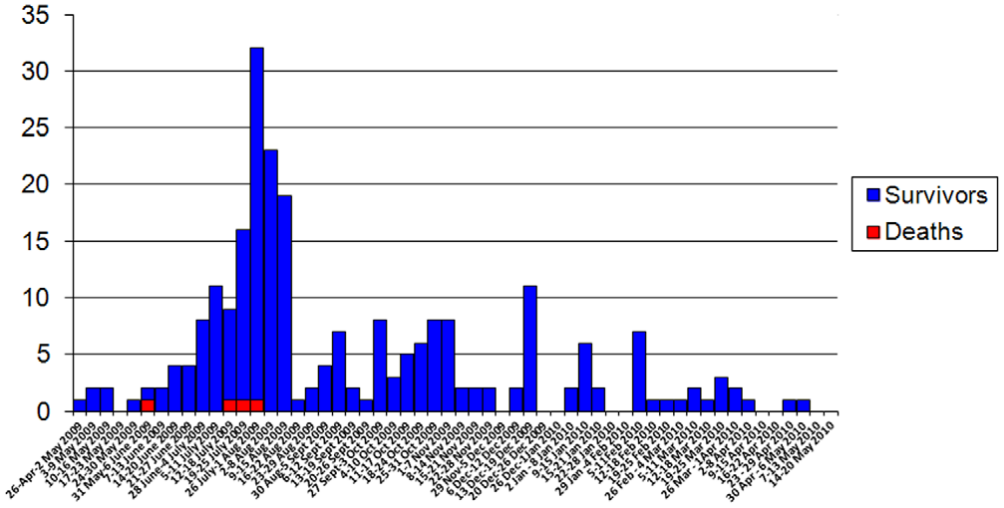
Cases and deaths from wet beriberi among AMISOM troops in Mogadishu, Somalia, by week of admission. n = 241.

From April 26, 2009–May 1, 2010, 241 patients evaluated at the level II hospital in Mogadishu met the case definition. Information and details of cases who presented to the hospital from April 26–August 19, 2009 (n = 136) were recorded by providers in Mogadishu and verbally communicated to the investigation team; information and details of cases who presented to the hospital from August 20, 2009–May 1, 2010 (n = 105) were more systematically collected and recorded by the outbreak investigation team. Cases first occurred during late April 2009, peaked in late July 2009, and continued to be detected until May 2010 (Figure 1). There were no cases identified from the surrounding community. Of the 241 case-patients, at least 52 (21.6%) were evacuated for further treatment in Kenya, Uganda, or Burundi; four patients (1.7%) died.

On August 18, 2009, after concluding that the patients' clinical signs and symptoms could be compatible with wet beriberi, a physician at the level II hospital in Mogadishu administered intramuscular thiamine to an ill soldier. This resulted in rapid and dramatic improvement of his condition within hours, including resolution of edema and cardiac symptoms. This treatment was repeated in other cases with similar success, and was quickly adopted as standard treatment for soldiers with identifying clinical symptoms. A hypothesis of thiamine deficiency resulting in wet beriberi was therefore confirmed. By September 1, 2009, thiamine supplementation was distributed to all troops (initially 10 mg tablets daily for two weeks, followed by 1 mg tablets daily for four weeks). Dietary improvements, including the introduction of a greater proportion of vegetables and meat in soldiers' diets, were also implemented. Although additional cases did occur after these interventions, the number and severity of beriberi cases decreased substantially over the next few months.

From the time of initiation of thiamine supplementation on 1 September 2009 until 1 May, 2010, eight severe case-patients requiring referral to hospitals outside the country occurred among the 103 cases identified (7.8%). There were no reported deaths. However, interviews revealed that the uptake of this intervention was variable; not all troops reported taking the recommended thiamine.

### Case Control Study

Among 136 cases identified on or before August 19, 126 (92.6%) were enrolled in the case-control study; 46 controls were also enrolled in the study. Cases were less likely than controls to report supplementing their diet with local food in the last two months [Odds Ratio (OR) 0.30; 95% Confidence Interval (CI) 0.12–0.72], and more likely to report participating in guard duties (OR 3.68; 95% CI 1.4–9.68) or attacks from enemy fire either on a daily basis (OR 13.8; 95% CI 4.5–42.4) or once a week (OR 6.5, 95% CI1.5–27.5). Age, rank, working hours, participation in recreation activities, and smoking and alcohol intake were not significantly different between cases and controls (Table 2).

**Table 2:** Results of case control study conducted August 19–21, 2009 in Mogadishu, Somalia.

Variables	Case (total = 126)	Controls(Total = 46)	OR(95% CI)	P-value
**Camp of residence**				
Statehouse	37(29.4)	12(26.7)	Ref	-------
Algezera 1	1(0.8)	11(24.4)	0.03(0.00,0.25)	0.001
Base Camp	18(14.3)	12(26.7)	0.49(0.18,1.29)	0.149
Immigration	3(2.4)	0(0.0)	—	—
K4	3(2.4)	1(2.2)	0.97(0.09,10.25)	0.982
Sea Port	40(31.8)	7(15.6)	1.85(0.66,5.21)	0.242
Shakalla	24(19.1)	2(4.4)	3.89(0.80,18.94)	0.092
**Age**				
20–29	64(50.8)	21(45.7)	Ref	------
30–39	55(43.7)	20(43.5)	0.90(0.44,1.84)	0.777
40–49	6(4.8)	3(6.5)	0.66(0.15,2.86)	0.575
> = 50	1(0.8)	2(4.4)	0.16(0.01,1.90)	0.148
**Rank**				
Junior Officers	2(1.6)	2(4.4)	0.29(0.04,2.14)	0.223
Senior Non Commissioned Officers	29(23.2)	16(35.6)	0.52(0.25,1.10)	0.086
Other Ranks	94(75.2)	27(60.0)	Ref	------
**Duration of stay in Mogadishu (Months)**				
3–5	91(72.2)	28(60.9)	Ref	-------
6–8	13(10.3)	6(13.0)	0.67(0.23,1.92)	0.452
9–11	22(17.5)	12(26.1)	0.56(0.25,1.28)	0.172
**History of fever in last 2 months**	58(46.0)	19(41.3)	1.21(0.61,2.40)	0.581
**Sleep under mosquito net**				
Not at all	22(17.6)	9(20.0)	Ref	------
Sometimes	72(57.6)	14(31.1)	2.10(0.80,5.52)	0.130
All the time	31(24.8)	22(48.9)	0.58(0.22,1.49)	0.255
**Eat local food in last two months**	12(9.5)	12(26.0)	0.30(0.12,0.72)	0.008*
**Working hours**				
> = 12	65(57.0)	21(56.8)	Ref	------
<12	49(43.0)	16(43.2)	0.99(0.47,2.09)	0.978
**Activities/Duties**				
Guard duties	86(82.7)	13(56.5)	3.68(1.40,9.68)	0.008*
Others	18(17.3)	10(43.5)	Ref	-------
**Participate in Recreational activities**	20(17.1)	9(21.4)	0.76(0.31,1.82)	0.533
**Perception of life under threat**				
Not under threat	12(9.5)	6(13.0)	Ref	------
under threat	114(90.5)	40(87.0)	1.43(0.50,4.05)	0.506
**Frequency of camp attack from enemy fire**				
Not at all	7(6.7)	14(33.3)	Ref	-----
Daily	69(86.4)	10(23.8)	13.80(4.49,42.46)	<0.001*
Once a week	13(12.5)	4(9.5)	6.50(1.54,27.49)	0.011*
Other	15(14.4)	14(33.3)	2.14(0.67,6.86)	0.199

*Statistically significant (p<0.05).

### Clinical investigation

Between June 1 and August 7, 2009, 44 AMISOM troops were referred to AKUH-N; of these 33 met the case definition. Twenty-nine patients recovered and were discharged from the hospital, and 4 died (Table 3). The mean age was 29 years (range 24–39 years). All patients had lower extremity edema and at least one of the following symptoms: palpitations (22/33; 60.6%); chest pain (14/33; 42.4%), difficulty breathing (14/33; 42.4%) and abdominal pain (11/33; 33.3%). Onset of symptoms for these patients ranged between 1–37 days prior to admission (median = 33 days). One patient was HIV-positive, but no other chronic illnesses were reported. For all 33 patients, complete blood counts and blood chemistries processed on admission were normal. Some cases had evidence of hepatic and renal abnormalities. Direct bilirubin was elevated in 21/33 (63.6%) of cases. Liver enzymes (SGOT, SGPT and GGT) were elevated in <20% of tested cases. Creatinine levels were elevated in13/33 cases (39.4%); of these, 4 cases also had high blood urea nitrogen (Table 1).

**Table 3:** Characteristics of beriberi cases admitted to the Aga Khan Hospital, Nairobi, Kenya (N = 33).

Demographics	N	Percent
Age Median(Range)	29(24–39)	
Sex(Male)	33	100%
**Country of origin**		
Burundi	9	27.3%
Uganda	22	66.7%
Days hospitalized, Median (Range)	6(1–38)	
**Outcome**		
Deaths	4	12.1%
**Presenting symptoms**		
Bilateral lower extremity edema	33	100.0
Palpitations	20	60.6
Chest Pain	14	42.4
Difficulty Breathing	14	42.4
Stomach Ache	11	33.3
Fever	10	30.3
Headache	10	30.3
Chills	9	27.3
Shortness of Breath	8	24.2
Muscle Pains	6	18.2
Joint Pains	6	18.2
Cough	4	12.1
Nausea	3	9.1
Vomiting	3	9.1
Sneezing	2	6.1
Sore Throat	1	3.0

On admission, 11/33 (33.3%) had tachycardia (pulse of >100 bpm). Of the 11 cases with tachycardia, nine had abnormalities on heart exam, including a systolic murmur (6/11) and a third heart sound (6/11). Of the 31 patients for whom echocardiography was performed, 6 (19.4%) had changes suggestive of right-sided heart failure, and 4/31 had changes consistent with pulmonary hypertension (right ventricular systolic pressure >35 mm Hg). Cardiac catheterization was done for 10 patients; no evidence of coronary artery disease was found.

Of the 33 cases, six were unstable during their hospital stay; five patients were transferred to the Intensive Care Unit (ICU) in cardio-respiratory failure and one patient was transferred to the High Dependency Unit (HDU) with symptoms suggestive of congestive right-sided heart failure.

Of the six patients requiring a higher level of cardio-respiratory care, four (from among the five ICU patients) died within 48 hours of admission. The fifth ICU patient and the HDU patient recovered after receiving supportive treatment, and both were discharged home.

Autopsies were done on two patients. Postmortem reports showed features suggestive of congestive cardiac failure. The first patient had generalized edema on inspection (pedal, sacral as well as facial edema), and evidence of pericardial effusion with increased left and right ventricular thickness. The lungs were edematous with areas of consolidation. Histology of lungs, liver, spleen, and kidneys showed extensive congestion. Postmortem examination of the second patient demonstrated congestion of the lungs with frothy edematous fluids noted; other organs were found to be grossly normal. Histology of the lung tissue showed lung edema and congestion.

### Laboratory Investigation

Testing for various infectious pathogens was performed for the first 18 cases admitted to AKUH-N. Twelve (67%) cases were positive for Hepatitis E; of these, nine had high direct bilirubin and two had elevated liver enzymes. IgM antibodies against dengue were detected in 11 (61%) cases. Samples were negative for Leptospira, coxsackie B, influenza virus and other viral, rickettsial and bacterial pathogens (Table 1). On November 9, 2009, blood samples taken from 16 cases prior to their treatment (drawn in September 2009) showed increased levels of erythrocyte transketolase activation coefficient in all samples, consistent with thiamine deficiency [Cobas mean = 1.34; range = 1.22 to 1.62 (normal = <1.25)]. Twenty-eight blood and 16 urine samples tested showed no evidence of argemone alkaloids.

### Environmental Investigation

Meals for troops were prepared in three different kitchens. Each kitchen prepared food for different dining halls that each served troops of certain ranks; senior officers, junior officers, and non-commissioned officers all ate at separate dining halls. Although all kitchens obtained ingredients from common stores, there was a greater proportion of meat and vegetables in the senior and junior officer kitchens compared to the non-commissioned officer kitchen. The food was prepared in a covered area, and cooked in large pots over an open fire. The meals consisted of polished rice, beans, polished maize flour, wheat flour, meat, powdered milk, tea, coffee, and occasional vegetables. In camps located in areas without major security incidents, troops reported occasionally buying supplemental food, such as chicken, locally.

Ten samples of cooking oil and seven cooked food samples that were collected from the common kitchen tested negative for argemone alkaloids (<6.5 ppb) at the Geneva Food Authority Control (Service de la Consommation et des Affaires Vétérinaires) in Geneva, Switzerland, the National Health Laboratory Service in Johannesburg, South Africa, and the US National Institute for Environmental Health Sciences in Columbus, Ohio, USA.

## Discussion

This report documents a large outbreak of beriberi due to thiamine deficiency among soldiers in Mogadishu exposed to a severely restricted diet. Although thiamine deficiency and beriberi have been common throughout history, large outbreaks of this type have been rare during the last 50 years.

Thiamine, or vitamin B1, is a water-soluble vitamin. The active form, thiamine pyrophosphate, functions as an essential coenzyme in the metabolism of carbohydrates. Body stores of thiamine are limited, and individuals subjected to a thiamine-poor diet can deplete body stores within one to three months [6]. The clinical syndrome associated with thiamine deficiency is called beriberi; it is often referred to as wet or dry, depending on whether cardiac or neurologic symptoms predominate. The predominant cardiac symptoms of wet beriberi are more common among young, physically active males [6] and are related to impaired carbohydrate metabolism resulting in lactic acidosis, leading to peripheral vasodilatation, high output cardiac failure, and pulmonary and peripheral edema [7]. In severe cases, rapid and fulminant cardiac collapse can occur, a rare syndrome called shoshin beriberi [8]. Although symptomatic thiamine deficiency occurs regularly in alcoholic patients with poor diets, outbreaks of beriberi are extremely rare and in modern times have been limited primarily to groups subjected to severely restricted diets, such as prison populations [9], detainees [10], and refugees [11].

The initial appearance of this outbreak presented a diagnostic quandary. Because of the unusual but very consistent characteristics of the presenting cases, a wide range of diagnoses were considered, including toxic ingestion, a variety of infectious diseases, inhalational injury, and several metabolic abnormalities. Based on the epidemiologic evidence, including an ongoing exposure to a monotonous and severely restricted diet, clinical evidence of rapid response to therapeutic thiamine administration, and laboratory evidence of increased erythrocyte transketolase activation coefficient, we believe that thiamine deficiency causing wet beriberi best explained this outbreak. Our case-control study, although limited by a small number of controls, supported the conclusion that the outbreak was related to diet: cases were significantly less likely than controls to have reported supplementing their diet with local foods obtained from the community in the two months prior to the case-control study. Cases were also more likely than controls to report feeling that their lives were constantly under threat, suggesting a risk of illness associated with poor security and likely decreased access to outside (locally-produced) sources of food.

Factors known to increase the risk of thiamine depletion include fever, which can increase metabolism; a diet which relies on polished rice as a primary source of carbohydrates; physical exertion, which raises the bodily requirements of thiamine; and a high ratio of carbohydrates to fat in the diet, which can increase thiamine utilization [6]. Thiamine is utilized in the metabolism of carbohydrates; for AMISOM troops, who consume high carbohydrate diets, thiamine requirements are high. Although the diet of the troops was not completely devoid of thiamine, we hypothesize that high thiamine requirements combined with a restricted diet increased the risk of thiamine deficiency and led to sporadic cases of beriberi among these troops. Mass supplementation of the troops with a multivitamin that included thiamine, and diversification of the diet, led to a pronounced decrease in new cases. Although additional cases did occur after our intervention was initiated, no further deaths were reported from this illness, and the outbreak gradually subsided.

The diagnosis of thiamine deficiency is often not straightforward, making the identification and management of thiamine-deficient patients difficult. The severity of thiamine deficiency disease is not always consistent with thiamine levels. During the investigation of an outbreak in a drug rehabilitation facility in Malaysia, testing of a large cohort of both affected and unaffected inmates (n = 154) revealed an equal prevalence of thiamine deficiency among symptomatic and asymptomatic inmates [12]. Even after supplementation for three weeks in deficient patients, thiamine levels may not normalize in some patients [13]. Further confusing the issue, thiamine depletion may be a marker for other micronutrient deficiencies, signaling a population at high risk for other nutritional problems. While multivitamin supplementation may improve the situation initially, dietary diversification is essential to ensure that any co-existing micronutrient deficiencies are corrected.

Our investigation is subject to several limitations. The investigation in Mogadishu was conducted amid extreme security constraints, which limited the access of the investigative team to the site of the outbreak. As a result, detailed dietary information, including the theoretical-thiamine content of a standard food ration, and the quantities of different menu items consumed by cases and controls, were not available for review and dose-response relationships could not be established. Similarly, detailed clinical and demographic information was not readily available for cases presenting before August 20, 2009, possibly limiting the completeness of our data. Data regarding neurological findings for cases in Mogadishu were not systematically collected or available for inclusion in this investigation, limiting out ability to document the range of neurologic involvement. However, for the 33 cases examined and followed in Nairobi, no neurologic deficits were documented. In addition, frequent routine troop rotations in Mogadishu likely affected the natural history of thiamine deficiency in the troops; the exposed population was not a single cohort. Healthy troops were rotated in and troops with months of exposure to the diet in Mogadishu were rotated out. These troop rotations further limited the availability of cases for follow up interviews, and we were not able to determine risk factors for illness among troops who became ill after the supplementation efforts began. Also, although testing on 16 cases showed increased levels of erythrocyte transketolase activation coefficient in all samples, consistent with thiamine deficiency, we did not test all cases, nor did we test controls.

Interestingly, a similar outbreak characterized by lower extremity edema, palpitations and dyspnea occurred among Somali frontier guards of the British East Africa Command in February 1942. Investigation revealed an inadequate diet, including “one orange per week” and a diet devoid of meat for two months. Rapid improvement was noted following therapeutic injection with thiamine, and the etiology of the outbreak was determined to be beriberi. Eighteen cases were identified in total, and the outbreak responded to dietary diversification [14]. Although evidence in the 1942 and the 2009–2010 outbreaks points clearly to the consumption of a thiamine-deficient diet, the recurrence of an extremely unusual event among Somalia-based troops warrants the consideration of the additional role, perhaps environmental in nature, of anti-thiamine factors [15]. In previous outbreaks, exposures related to the ingestion of thiaminase-containing products (e.g., betel nut, shellfish, poorly-cooked fish, substances high in tannin, and locally grown plants such as ferns) have been implicated in thiamine deficiency and beriberi [6]. Additionally, colonization of the intestines by thiaminase bacteria can lower thiamine levels by destroying ingested thiamine despite adequate dietary consumption [16]. Among AMISOM troops in Mogadishu, we were not able to elicit a history of exposure to anti-thiamine factors which could explain this outbreak.

Historically, very large outbreaks of beriberi occurred in Japan and have been hypothesized to be due to Citreoviridin, a mycotoxin produced by molds common in rice and which is known to cause a syndrome similar to wet beriberi in experimental animals [17]. The affected rice can be discolored (brown or yellow, depending on the mold). Elimination of beriberi in Japan was attributed to improvements in rice quality. Recent investigations of cases of beriberi in Brazil have supported a link to rice, and led to additional calls for improving rice quality [18], but the role of citreoviridin in these cases is disputed [19]. In Mogadishu, we did not note any discoloration of the stored rice, although we did not evaluate fungal colonization of rice samples. Once the diagnosis of beriberi was suspected, we did note a general improvement in food quality, which may have led to changes in environmental exposures during the course of this outbreak.

This outbreak of beriberi due to thiamine deficiency occurred in one of the most unstable and insecure settings in the world. The near constant attacks on AMISOM troops have resulted in a relative confinement of troops, and a reduction in the informal supplementation of their diets with locally acquired food items. Although beriberi is a preventable illness, this event must be viewed within the context of the operational and logistical challenges inherent in this environment. This outbreak highlights the need for ongoing nutritional vigilance among groups that are unable to acquire food independently, and that rely entirely on the provision of nutrition by outside sources.
